# Metabolic classification of bladder cancer based on multi-omics integrated analysis to predict patient prognosis and treatment response

**DOI:** 10.1186/s12967-021-02865-8

**Published:** 2021-05-13

**Authors:** Chaozhi Tang, Meng Yu, Jiakang Ma, Yuyan Zhu

**Affiliations:** 1 Department of Urology, The First Hospital of China Medical University, Shenyang, 110001 Liaoning Province China; 2 Department of Laboratory Animal Science, China Medical University, Shenyang, 110122 China; 3 Key Laboratory of Transgenetic Animal Research, Liaoning Province, China Medical University, Shenyang, 110122 China; 4 Department of Oncology, The Second Affiliated Hospital of Zhengzhou University, Zhengzhou, 450014 China

**Keywords:** Metabolic classification, Bladder cancer, Prognosis, Immunotherapy, Chemotherapy

## Abstract

**Background:**

Currently, no molecular classification is established for bladder cancer based on metabolic characteristics. Therefore, we conducted a comprehensive analysis of bladder cancer metabolism-related genes using multiple publicly available datasets and aimed to identify subtypes according to distinctive metabolic characteristics.

**Methods:**

RNA-sequencing data of The Cancer Genome Atlas were subjected to non-negative matrix fractionation to classify bladder cancer according to metabolism-related gene expression; Gene Expression Omnibus and ArrayExpress datasets were used as validation cohorts. The sensitivity of metabolic types to predicted immunotherapy and chemotherapy was assessed. Kaplan–Meier curves were plotted to assess patient survival. Differentially expressed genes between subtypes were identified using edgeR. The differences among identified subtypes were compared using the Kruskal–Wallis non-parametric test. To better clarify the subtypes of bladder cancer, their relationship with clinical characteristics was examined using the Fisher’s test. We also constructed a risk prediction model using the random survival forest method to analyze right-censored survival data based on key metabolic genes. To identify genes of prognostic significance, univariate Cox regression, lasso analysis, and multivariate regression were performed sequentially.

**Results:**

Three bladder cancer subtypes were identified according to the expression of metabolism-related genes. The M1 subtype was characterized by high metabolic activity, low immunogenicity, and better prognosis. M2 exhibited moderate metabolic activity, high immunogenicity, and the worst prognosis. M3 was associated with low metabolic activity, low immunogenicity, and poor prognosis. M1 showed the best predicted response to immunotherapy, whereas patients with M1 were predicted to be the least sensitive to cisplatin. By contrast, M2 showed the worst predicted response to immunotherapy but was predicted to be more sensitive to cisplatin, doxorubicin, and other first-line anticancer drugs. M3 was the most sensitive to gemcitabine. The risk model based on metabolic genes effectively predicted the prognosis of bladder cancer patients.

**Conclusions:**

Metabolic classification of bladder cancer has potential clinical value and therapeutic feasibility by inhibiting the associated pathways. This classification can provide valuable insights for developing precise bladder cancer treatment.

**Supplementary Information:**

The online version contains supplementary material available at 10.1186/s12967-021-02865-8.

## Background

Bladder cancer generally develops in the epithelial cells of the bladder and is the fifth most common type of cancer occurring worldwide, with 430 000 new cases and more than 165 000 deaths reported each year [1–3]. Particularly, muscle-invasive bladder cancer (MIBC) is associated with high morbidity and mortality owing to its heterogeneity and invasiveness, and represents the ultimate challenge in the diagnosis, treatment, and care of bladder cancer [4]. Robertson et al. [5] conducted a comprehensive genomic analysis of MIBC samples and mapped the molecular typing atlas of MIBC. Based on RNA-sequencing (RNA-seq) data, the following five molecular subtypes were identified: luminal-papillary, luminal-infiltrated, luminal, basal-squamous, and neuronal. The luminal-papillary subtype (35%) is characterized by the existence of *FGFR3* mutations and a low carcinoma in situ score; thus, the risk of progression is low, but the probability of response to cisplatin-based neoadjuvant chemotherapy is also low. The frequency of *FGFR3* mutation changes observed in luminal papillary tumors suggests that FGFR3 tyrosine kinase inhibitors may be an effective treatment. The intraluminal-infiltrated subtype showed a response to immune checkpoint therapy with atezolizumab in patients with metastatic or unresectable bladder cancer [6]. The basal-squamous subtype is characterized by a high expression of CD274 (PD-L1) and CTLA4 immune markers, and other signs of immune infiltration; thus, cisplatin-based neoadjuvant chemotherapy and immune checkpoint therapy are suitable treatment options for this subtype [7]. However, there is currently no clear targeted therapy available for the luminal subtype. The neuronal subtype is associated with the worst prognosis for patients with MIBC, which is characterized by the expression of neuroendocrine and neural markers; etoposide-cisplatin therapy is recommended as neoadjuvant and metastatic treatment. Establishment of this molecular classification combined with pathological morphology and molecular characteristics has provided further understanding of the pathogenesis and heterogeneity of bladder cancer, along with new insights and opportunities for prognostic application evaluation, disease monitoring, and personalized treatment.

In addition to the immune and molecular characteristics outlined above, the development and progression of cancer are characterized by a unique reprogramming of energy metabolism, which is necessary for the maintenance of highly proliferating cancer cells [8]. Recent studies have reported that bladder cancer cases can present a variety of characteristic metabolic changes, including increased aerobic glycolysis, increased de novo fat synthesis, glutamine consumption, and oxidative metabolism imbalance, which collectively contribute to the rapid growth and proliferation of tumor cells by providing energy and raw materials for biomacromolecule synthesis [9]. Therefore, an in-depth study of the metabolic characteristics and regulatory mechanisms of bladder cancer is essential for the development of agents that can target tumor metabolism. Although recent advances in high-throughput genomic bioinformatics analysis have provided the platforms and opportunities for the discovery of new bladder cancer biomarkers and metabolic targets, there is currently a lack of molecular typing studies focusing on the metabolic characteristics of bladder cancer.

Therefore, in this study, we performed a comprehensive analysis of bladder cancer metabolism-related genes using multiple publicly available datasets, with the goal of identifying subtypes according to distinctive metabolic characteristics. We further compared the prognostic characteristics, clinical characteristics, immune infiltration, genetic variation, chemotherapy and immunotherapy response, and other aspects to comprehensively elucidate the characterization of the proposed metabolic subtypes of bladder cancer. These results can provide new knowledge and act as a supplement for the molecular subtyping of bladder cancer from the perspective of metabolic regulation.

## Methods

### Data processing

The datasets used to identify the metabolic subtypes of bladder cancer were obtained from The Cancer Genome Atlas (TCGA), Gene Expression Omnibus (GEO), and ArrayExpress databases. RNA-seq data (FPKM) of 19 normal samples and 414 cancer samples were downloaded from TCGA Knowledge Base (https://portal.gdc.cancer.gov/repository), and gene annotation was performed using the Ensemble database. The ArrayExpress database contains FPKM RNA-seq and clinical data (N = 476) of 476 cases of early urothelial carcinoma (E-MTAB-4321) from the European Genome-Phenome Archive. The expression matrices of the four GEO datasets GSE13507 (N = 165), GSE32548 (N = 131), GSE31684 (N = 93), and GSE32894 (N = 308) were quantile-normalized, and the genes were annotated in their respective platform files: Illumina human-6 v2·0 expression BeadChip, Illumina HumanHT-12 v3·0 expression BeadChip, [HG-U133_Plus_2] Affymetrix Human Genome U133 Plus 2·0 Array, and Illumina HumanHT-12 v3·0 expression BeadChip, respectively. Information regarding the molecular classification of bladder cancer from TCGA and LUND University can be found in the supplementary documents of Robertson [5] and Sjodahl [10].

### Identification of new bladder cancer subtypes based on metabolic genes

According to the previously published 2752 metabolism-related genes encoding all known human metabolic and transport enzymes [11], genes with a median expression level below 0.5 in all bladder cancer samples were excluded, resulting in a matrix of 1734 metabolism-related genes for analysis. For TCGA discovery cohort, non-negative matrix factorization (NMF) was used for unsupervised decomposition and clustering. NMF clustering was further performed with the E-MTAB-4321 and GSE32984 datasets as validation cohorts using the same genes. Gene functional enrichment analysis was performed using Gene Ontology (GO) [12], Kyoto Encyclopedia of Genes and Genomes (KEGG) [13], and Metascape [14].

### Estimation of immune infiltration and matrix composition

CIBERSORT is a deconvolution method based on gene expression profile data to estimate the absolute abundance of 22 human immune cell populations [15]. We also collected the gene set of human matrix components through the relevant literature and used single-sample gene set enrichment analysis (ssGSEA) as an additional method to calculate an enrichment score, which represents the absolute degree of enrichment of the gene set in each sample of the given datasets. In addition, the ESTIMATE algorithm [16] was used to calculate the immune and stromal scores in each sample, as well as tumor purity. The gene sets used to assess hypoxia status were based on previous studies [17–21].

### Gene set variation analysis (GSVA)

GSVA is an expression matrix that takes a single gene as a feature and converts it into an expression matrix that uses a specific gene set as a feature. This unsupervised algorithm was then used to calculate the non-parametric enrichment score of a specific gene set in each sample. The gene sets related to sugar metabolism, lipid metabolism, and amino acid metabolism were obtained from GSEA c2.cp.kegg.v7.0.symbols.gmt, which was used to compare the differences in metabolism between subtypes that were statistically evaluated using the Kruskal–Wallis test.

### Immunotherapy response prediction

Tumor Immune Dysfunction and Rejection (TIDE) [22] is a new computing architecture that integrates data on two tumor immune escape mechanisms. The result is considered to be a substitute for a single biomarker to effectively predict the effect of immune checkpoint suppression therapy. We used TCGA expression data to predict the differences in the response to immunotherapy for each bladder cancer subtype and the cell types that affect T cell infiltration in tumors, including cancer-associated fibroblasts, myeloid-derived suppressor cells, and tumor-associated M2 macrophages.

### Chemotherapy response prediction

Based on the largest available public pharmacogenomics database [Genomics of Cancer Drug Sensitivity (GDSC), https://www.cancerrxgene.org/], we used TCGA FPKM RNA-seq expression profile to predict the chemical reaction of each sample. The prediction process was carried out using the R package ‘pRRophetic’, in which the half-maximal inhibitory concentration (IC50) was estimated using ridge regression for the sample, and the accuracy of the prediction was evaluated 10 times and cross-validated according to the GDSC training set. All parameters were set to default values.

### Statistical analysis

Survival of patients with different metabolic subtypes of bladder cancer was compared by plotting Kaplan–Meier curves and was analyzed using the log-rank test. Differentially expressed genes between subtypes were identified using edgeR according to a |log2 fold change|> 1 and P < 0·05 as the screening threshold parameters. The Kruskal–Wallis non-parametric test was used to compare differences among identified subtypes. To better clarify the subtypes of bladder cancer, their relationship with clinical characteristics was evaluated by performing the Fisher’s test. To exclude false positives, associations were strictly screened according to a false discovery rate < 0.05 and verified by multiple datasets. To construct a risk prediction model, we used the random survival forest method to analyze right-censored survival data based on key metabolic genes. Random survival forest is based on the retention principle of survival forests, which defines overall mortality as a simple and interpretable mortality measure that can be used as a predictive result. Moreover, construction of an integrated model based on the decision tree can considerably improve the prediction performance. We used the randomForestSRC R package for this purpose. Univariate Cox regression, lasso analysis, and multivariate regression were then used sequentially to identify genes of prognostic significance. All calculations and statistical analyses were conducted using R (version 3.5.3), and all tests were two-sided; P < 0.05 was considered statistically significant.

## Results

### Identification of metabolic subtypes of bladder cancer based on NMF

**Fig. 1:**
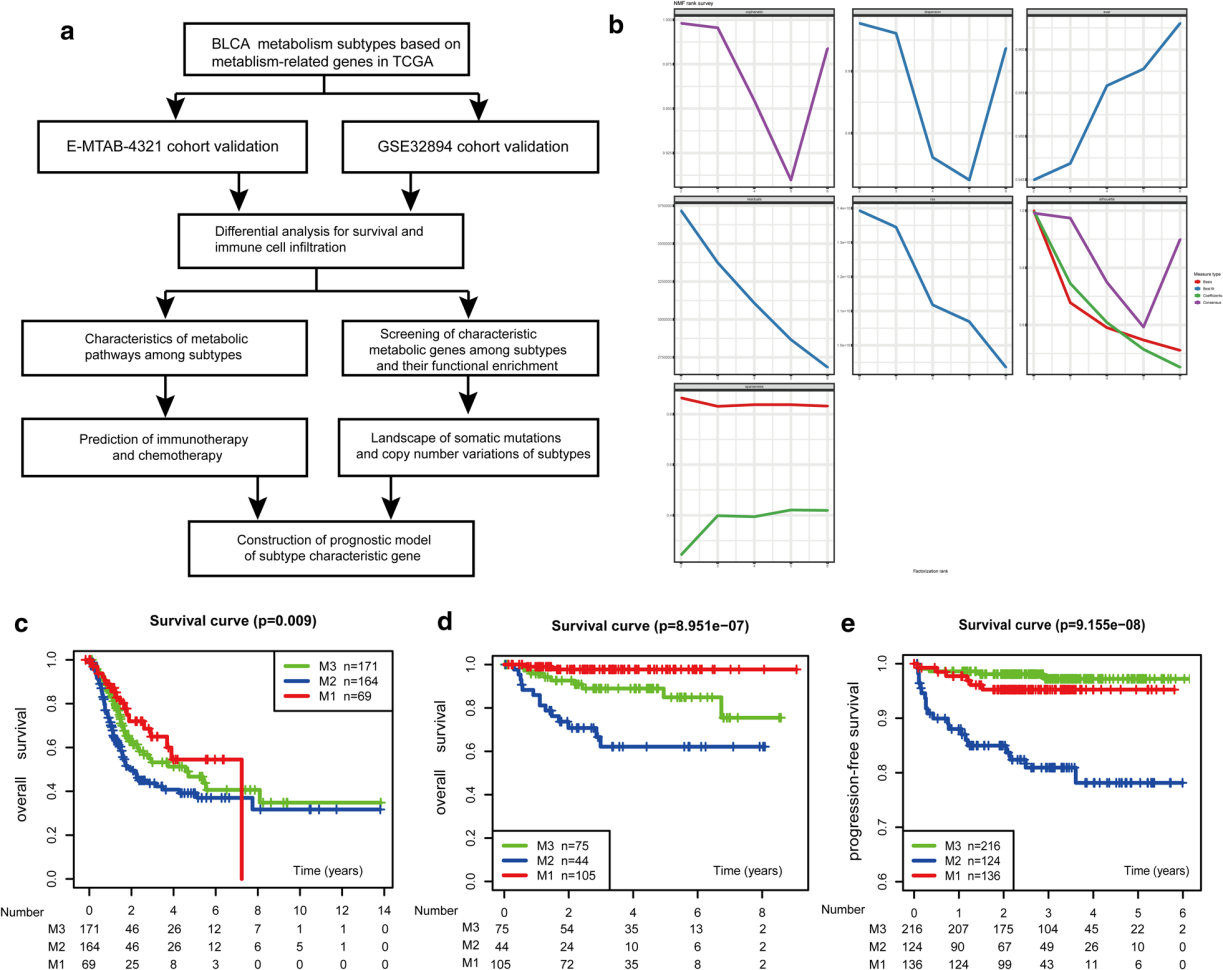
NMF consensus clustering performed to identify bladder cancer (BLCA) subclasses. **a** Workflow schematic. **b** NMF clustering performed using 1734 metabolism-related genes in TCGA cohort, along with the co-location correlation coefficient, bias, and best fit for k = 2–6. **c** Overall survival (OS) of TCGA cohort. **d** OS of the GSE32894 cohort. **e** Progression-free survival (PFS) of the E-MTAB-4321 cohort

For conducting NMF analysis, 2752 human metabolism-related genes were selected based on previous reports [11]. After removing the data on metabolism-related genes with low expression abundance in TCGA cohort, data on a total of 1734 metabolism-related genes were obtained for the cluster analysis. The same 1734 metabolism-related genes were considered in NMF-based cluster analysis of the two validation cohorts GSE32984 and E-MTAB-4321 (Fig. 1a). The comprehensive clustering results of the three cohorts were considered, and K = 3 was determined to be the best clustering number (Fig. 1b and Additional file 1: Figure S1). Based on the expression levels of metabolism-related genes, samples were sequentially classified into the M1, M2, and M3 subcategories of bladder cancer. A prognostic difference based on overall survival (OS) or progression-free survival (PFS) was observed according to this subtype classification in all three datasets (TCGA-OS, P = 0.009; GSE32894-OS, P < 0.001; E-MTAB-4321-PFS, P < 0·001). In TCGA cohort, patients with the M1 subtype (median survival = 536 days) had a better prognosis than those with the M2 (median survival = 423 days) and M3 (median survival = 483 days) subtypes. Similar results were found for the GSE32894 and E-MTAB-4321 validation cohorts (Fig. 1c).

### Characteristic genes and potential regulatory pathways of metabolic subtypes of bladder cancer

To better characterize the classification of the metabolic subtypes of bladder cancer, we used paired differential expression levels to construct a heat map of 289 subtype-specific upregulated genes (P < 0.05 and |log2 fold change|> 1), including 77, 257, and 48 characteristic genes of the M1, M2, and M3 subtypes, respectively. Functional enrichment analysis with GO and KEGG showed significant differences in the top-ranked pathways among the subtypes. The M1 subtype was related to processes such as drug metabolism-cytochrome P450, chemical carcinogenesis, glutathione metabolism, platinum drug resistance, and hormone metabolism; the M2 subtype was associated with drug metabolism-cytochrome P450, chemical carcinogenesis, arachidonic acid metabolism, alpha-linoleic acid metabolism, linoleic acid metabolism, ferroptosis, and butanoate metabolism; and the M3 subtype-associated genes were mainly involved in metabolism-cytochrome P450, chemical carcinogenesis, tyrosine metabolism, and hormone regulation (Additional file 2: Figure S2).

### Verification of metabolism-related signatures of bladder cancer subtypes

**Fig. 2:**
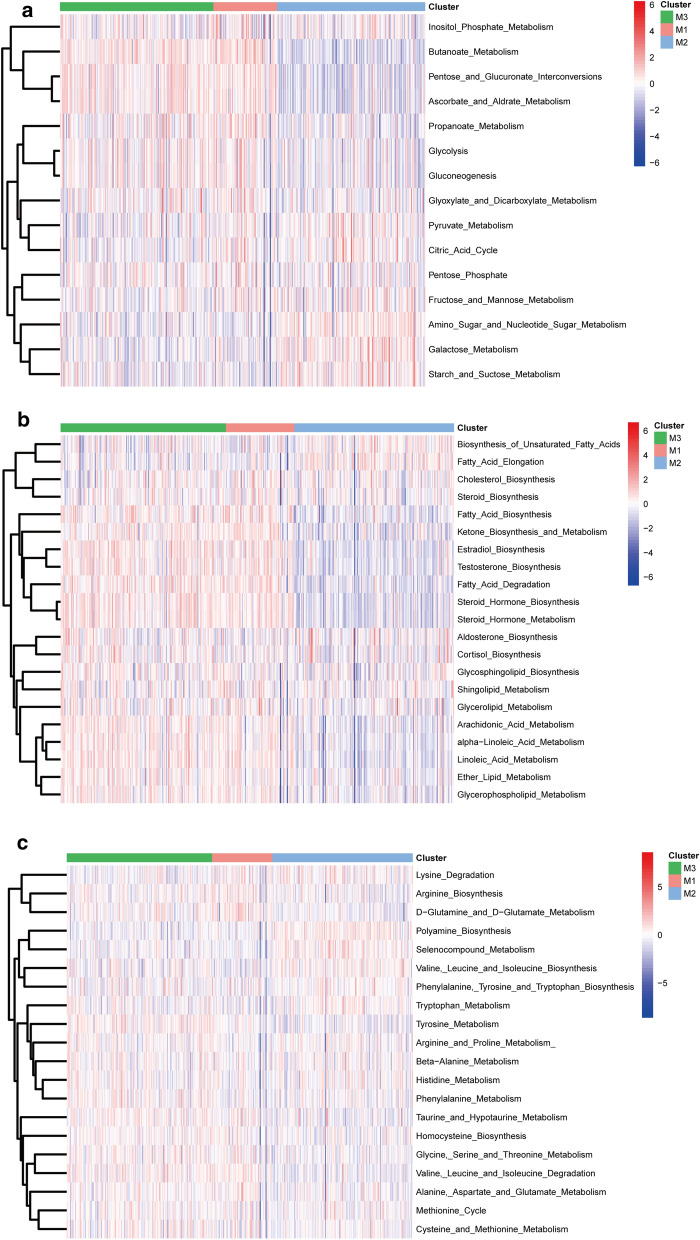
Characteristic metabolic pathways observed among bladder cancer subtypes. **a** glucose metabolism, **b** lipid metabolism, and **c** amino acid metabolism

The ssGSEA algorithm was used to quantify a total of 115 metabolic processes, focusing on the different characteristics of the three major types of metabolism (sugar, lipid, and amino acid metabolism) in the subtypes. Among the specific pathways involved in glucose metabolism, M1 and M3 were significantly associated with activated butanoate metabolism, pentose and glucuronate interconversions, and ascorbate and aldarate metabolism. M2 was associated with activated amino sugar and nucleotide sugar metabolism, galactose metabolism, and starch metabolism. Similarly, M1 and M3 appear to be related to the synthesis and metabolism of fatty acids and ketone hormones, whereas M2 showed upregulation in fatty acid modification and other pathways. In the regulatory pathways involving amino acid metabolism, M2 was associated with enhanced activation of the synthesis and metabolism of polyamine biosynthesis, seleno metabolism, and tryptophan (Fig. 2).

### Correlation between metabolic subtypes of bladder cancer and immune infiltration

**Fig. 3:**
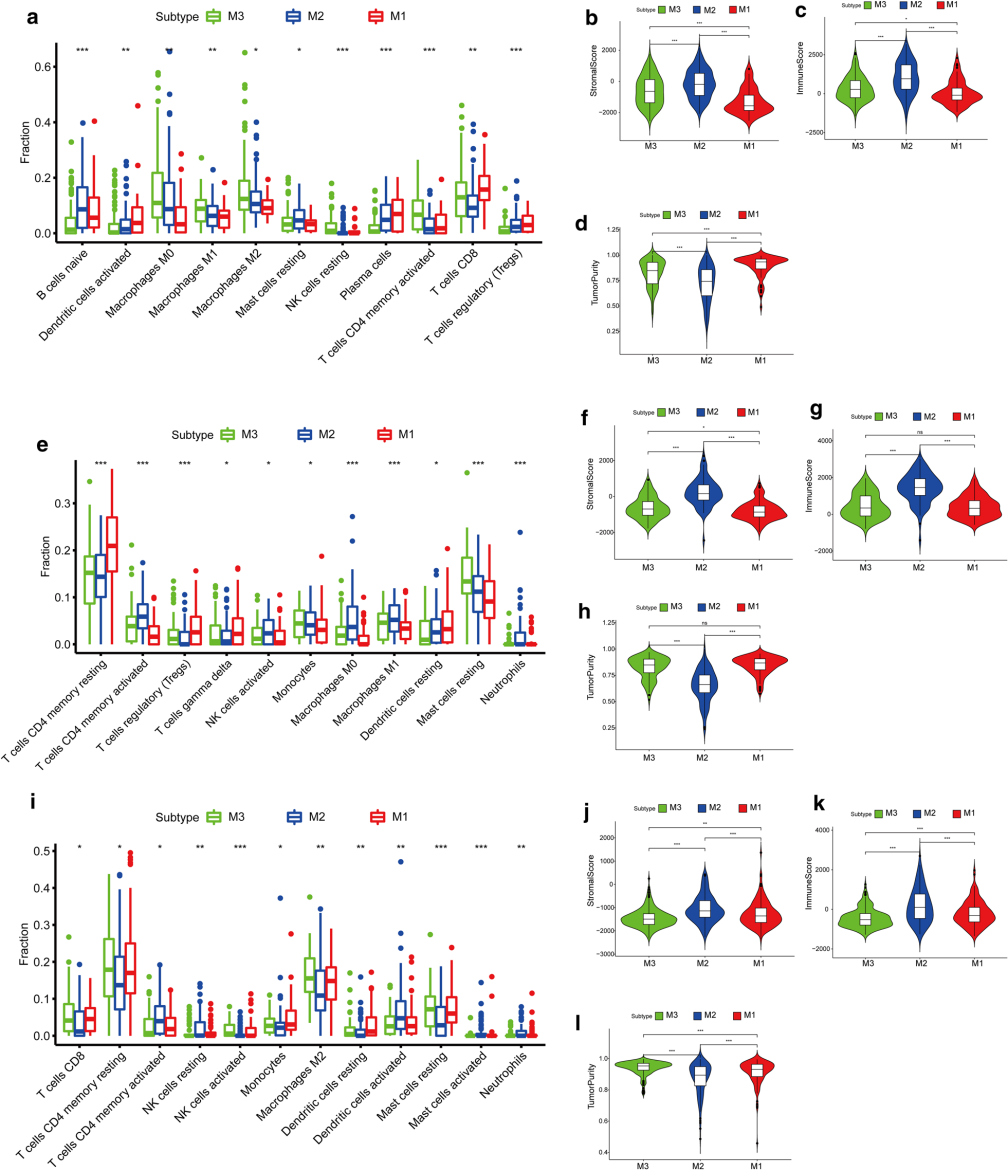
Immune infiltration among bladder cancer subtypes. **a** Immune cell components that differ between subtypes in TCGA cohort. **b**–**d** Immune, stromal score, and tumor purity between subtypes in TCGA cohort. **e** Immune cell components that differ between subtypes in the GSE32894 cohort. **f**–**h** Immune, stromal score, and tumor purity between subtypes in the GSE32894 cohort. **i** Immune cell components with differences between subtypes in the E-MTAB-4321 cohort. **k**–**l** Immune, stromal score, and tumor purity between subtypes in the E-MTAB-4321 cohort. *P < 0.05, **P < 0.01, ***P < 0.001; *ns* not significant

**Fig. 4:**
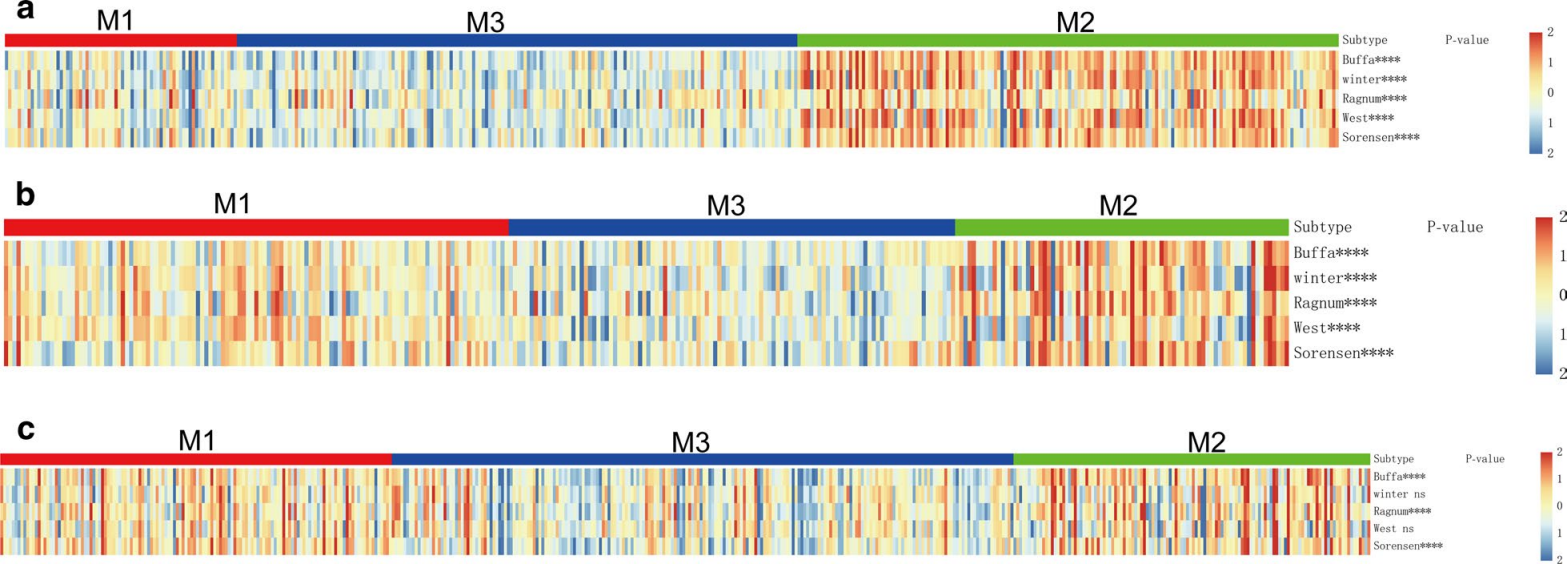
Hypoxia status observed among bladder cancer subtypes. Profiles of hypoxia gene sets in **a** TCGA, **b** GSE32894, and **c** E-MTAB-4321 cohorts. ****P < 0.0001; *ns* not significant

The ESTIMATE algorithm [16] was used to calculate the stromal and immune scores between each subtype, as well as tumor purity to characterize the regulatory relationship between the metabolic subtypes of bladder cancer and the tumor microenvironment. As shown in Fig. 3, the stromal and immune scores of the bladder cancer subtypes were ranked from low to high according to M1 < M3 < M2, which is consistent with their hypoxia status (Fig. 4), and the tumor purity showed the opposite trend. This result matched the trend related to differences in prognosis among subtypes. The CIBERSORT algorithm [15] results showed that among the 11 immune cell subpopulations with significant differences among subtypes, the M1 subtype was associated with activated dendritic cells, plasma cells, CD8 T cells, and regulatory T cells, representing a significant increase of these cell populations compared with the other subtypes. The M2 subtype showed a significant increase in naive B cells and resting mast cells compared to the other subtypes, whereas the populations of resting natural killer cells, activated CD4 memory T cells, and CD8 T cells had the lowest abundance in M2. The M3 subtype had the highest abundance of M0, M1, and M2 macrophages.

### Correlation between metabolic subclasses and clinical features of bladder cancer patients

The proportions of samples in the T stage in the TNM system differed significantly among the three subtypes. In TCGA cohort, there were differences among subtypes according to tumor grading, disease stage, M stage, and molecular subtypes. The validation sets GSE32894 and E-MTAB-4321 also showed significant differences in sex, tumor grade, molecular subtype, tumor grading, tumor size, histology, and cancer in situ in the disease course among metabolic subtypes of bladder cancer (Additional file 6: Table S1, Table S2 and Table S3).

### Sensitivity of immunotherapy and chemotherapy among metabolic subclasses of bladder cancer

**Fig. 5:**
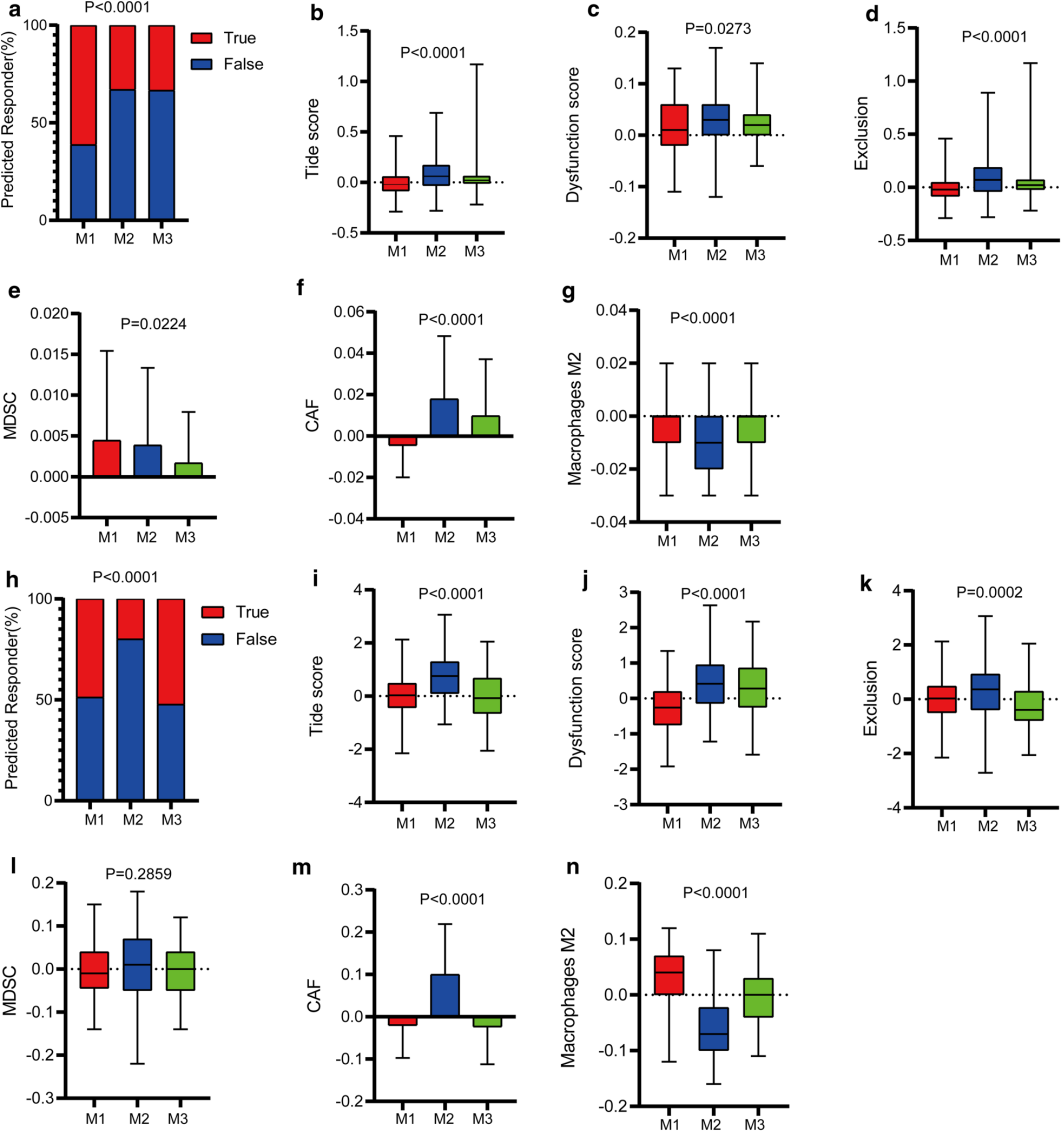
Immunotherapy prediction using the TIDE algorithm. **a** Differences in response to immunotherapy among subtypes; **b** TIDE score, **c** dysfunction score, **d** exclusion score, **e** myeloid-derived suppressor cell (MDSC), **f** cancer-associated fibroblast (CAF), and **g** M2 macrophages score in TCGA cohort. **h** Differences in response to immunotherapy among subtypes; **i** TIDE score, **j** dysfunction score, **k** exclusion score, **l** MDSC, **m** CAF, and **n** M2 macrophages score in the GSE32894 cohort

Based on the TIDE algorithm, M1 was predicted to be much more responsive to immunotherapy than M2 and M3, and M2 had a higher TIDE score than the other subtypes. Among the cell types that limited the infiltration of T cells in tumors, the M2 subtype included cancer-associated fibroblasts, myeloid-derived suppressor cells, and tumor-associated macrophages, indicating greater ability of cytotoxic T cells to kill cancer cells to a certain extent in this subtype. However, the M2 subtype also showed a high degree of T cell dysfunction based on the dysfunction score (Fig. 5). These results reflect the strong immune escape characteristics of the M2 subtype of bladder cancer compared with the other subtypes.

**Fig. 6:**
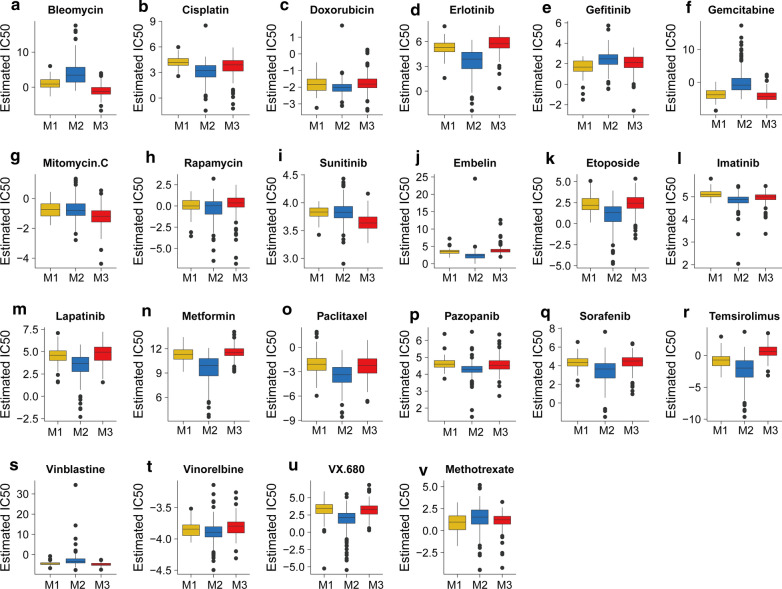
Chemosensitivity prediction among subtypes of bladder cancer in TCGA cohort. Chemosensitivity prediction among subtypes of bladder cancer in TCGA cohort based on the chemical drug sensitivity of the GDSC database. IC50, half-maximal inhibitory concentration

Using the GDSC database, among all first-line, second-line, or other reported drugs for bladder cancer, M1 was predicted to be the most sensitive to gefitinib and methotrexate; M3 was predicted to be the most sensitive to bleomycin, gemcitabine, mitomycin C, sunitinib, and vinblastine; and M2 was predicted to be the most sensitive to cisplatin. Similar sensitivity of the three subtypes was found for the other 15 drugs (Fig. 6 and Additional file 6: Table S4).

### Landscape of somatic mutation and copy number variation of metabolic subtypes of bladder cancer

**Fig. 7:**
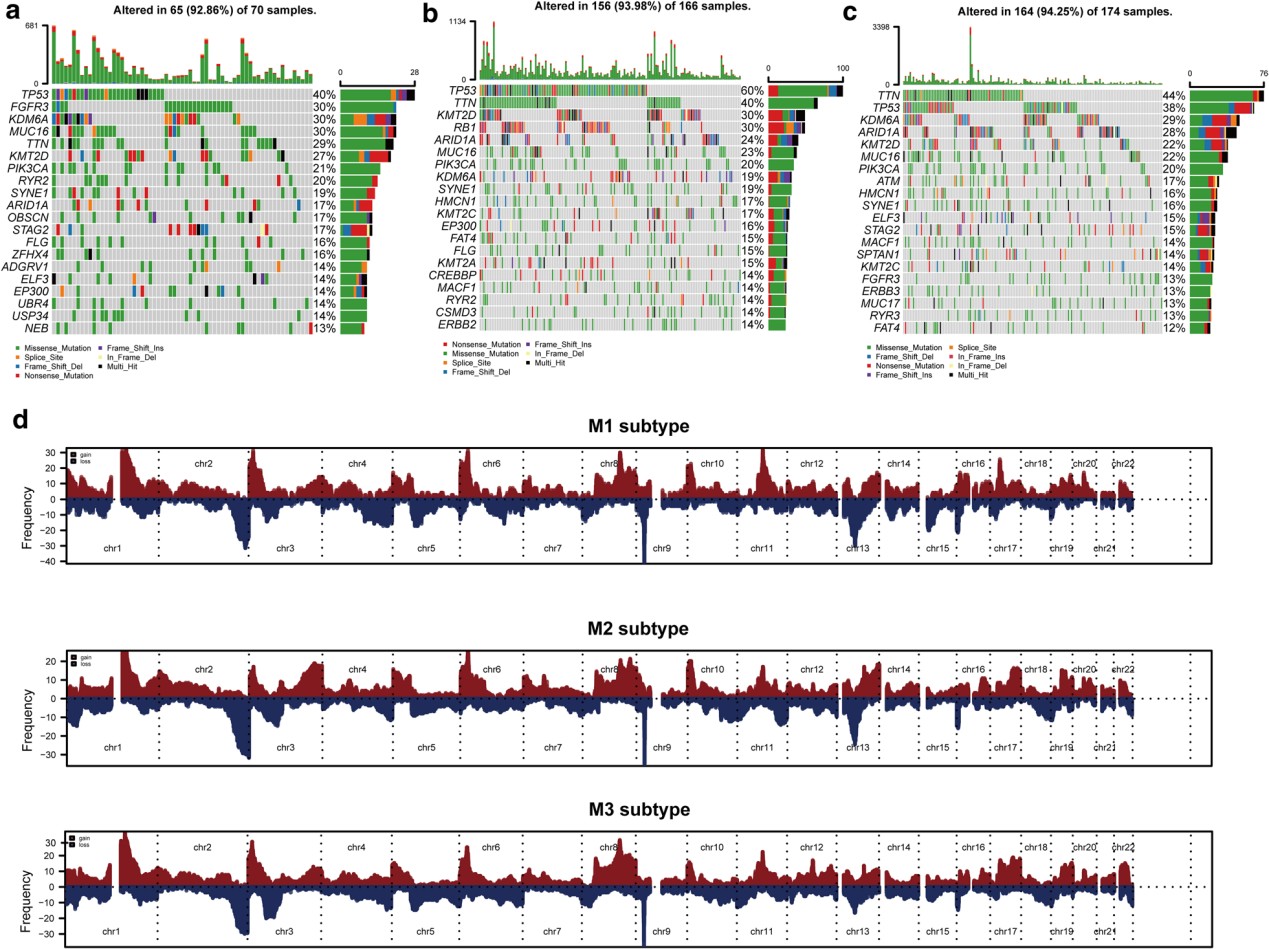
Landscape of somatic mutation and copy number variation of bladder cancer subtypes. Waterfall plots of bladder cancer somatic mutations for the **a** M1, **b** M2, and **c** M3 subtypes. **d** Distribution map of copy number variations of the three subtypes in 22 human autosomes

To further reveal the genomic differences among metabolic subtypes of bladder cancer and identify meaningful somatic mutations, we analyzed the top 20 genes with mutation frequencies exceeding 10% in samples of each subtype, which are displayed as a waterfall plot in Fig. 7. *SYNE1, KMT2D, PIK3CA, TP53, MUC16, ARID1A, KDM6A*, and *TTN* were in the top 20 genes of all three subtypes. Among them, *TP53* contributed to 40%, 60%, and 44% of the total mutation frequency in M1, M2, and M3, respectively. The mutation frequencies of *ARID1A* and *TTN* were increased in M2 (24% and 40%) and M3 (28% and 44%) compared with those of M1 (17% and 29%), and the mutation frequency of *MUC16* was higher in M1 (30%) than in M2 (23%) and M3 (22%). The mutation frequencies of *SYNE1* and *PIK3CA* were not significantly different among M1 (19% and 21%), M2 (19% and 20%), and M3 (16% and 20%). There was a trend of an increase in the mutation frequencies of *KDM6A* and *KMT6A* in M1 (30% and 27%), M2 (19% and 30%), and M3 (29% and 22%) (Fig. 6a–c).

Metascape biological function enrichment analysis showed that the top 20 mutant genes of the M1, M2, and M3 subtypes were involved in the development process, metabolism, and immune process (Additional file 3: Figure S3a–c), with differences in the central regulating genes among subtypes (Additional file 3: Figure S3g–i). The mutant genes of M1 and M3 were more associated with bladder cancer in situ and partially invasive bladder cancer, whereas the mutant genes of M2 were more strongly related to transitional cell carcinoma; this difference also largely corresponds to the differences in the prognosis of each subtype (Additional file 3: Figure S3j–l). Compared with the M1 subtype, the M2 and M3 subtypes showed higher similarity in chromosomal aberrations (Fig. 6d and Additional file 4: Figure S4). Specifically, the somatic chromosomes of the tumors in the M2 and M3 subtypes had several and similar aberrated (amplified or missing) sites. By contrast, there were few somatic chromosomal aberration sites of tumors in M1 samples, which were largely concentrated at certain regions and of low frequency. Among them, the rates of censored oncogenes in the M1, M2, and M3 subtypes were 28.57%, 30.91%, and 33.14% for *CDKN2A* (9p21.3); 28.57%, 30.3%, and 31.97% for *CKKN2B* (9p21.3); 27.14%, 24.24%, and 27.33% for *MTAP* (9p21.3); and 11.43%, 8.48%, and 8.14% for *RB1* (13q14.2), respectively. The amplification rates of oncogenes in the M1, M2, and M3 subtypes were 18.57%, 15.15%, and 13.95% for *E2F3* (6p22.3), and were 15.71%, 8.48%, and 16.86% for *DDR2* (1q23.3), respectively.

### Prognostic risk model based on characteristic genes of metabolic subtypes of bladder cancer

**Fig. 8:**
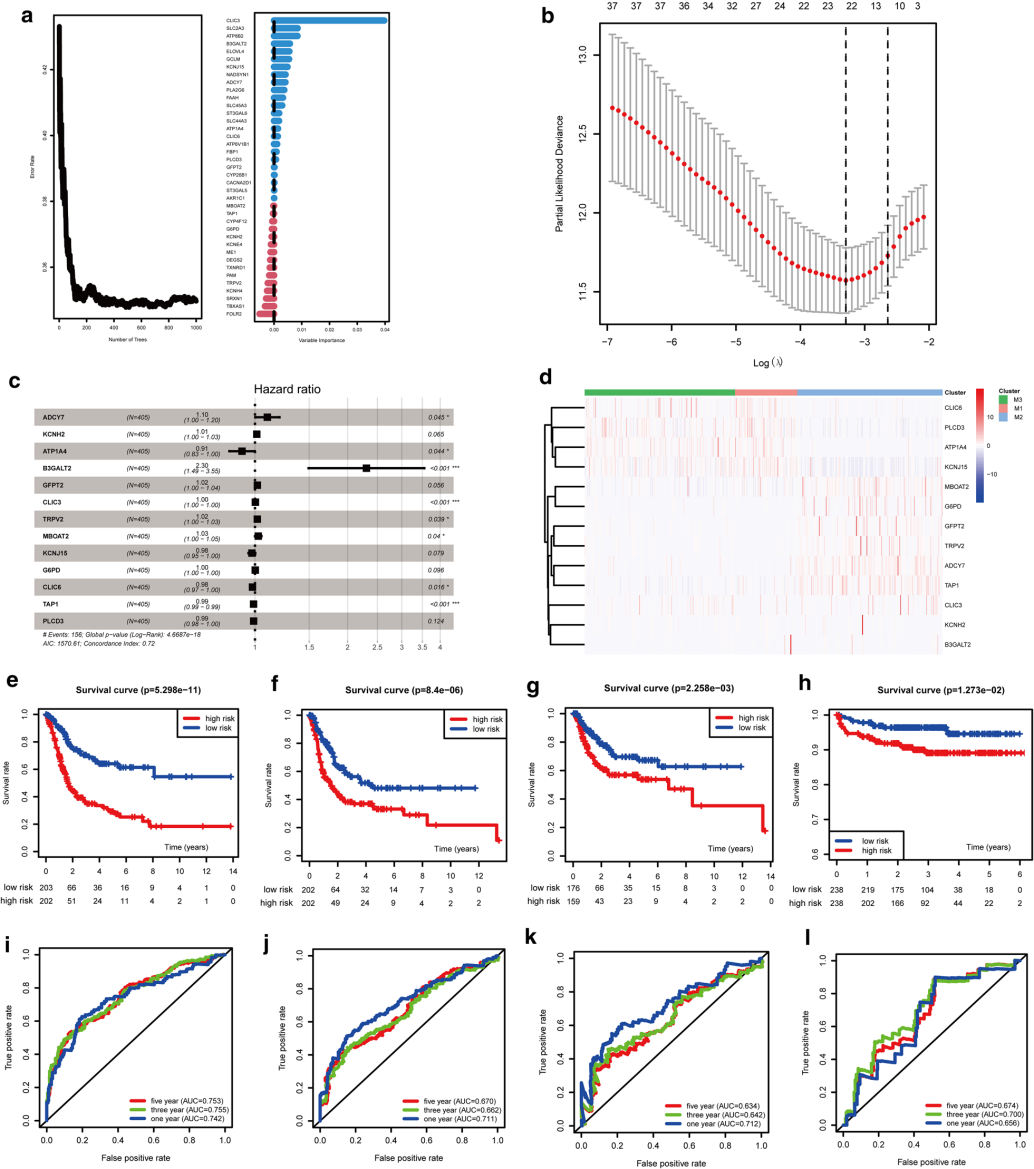
Prognostic risk model based on characteristic genes of the metabolic subtypes. **a** Random survival forest analysis of characteristic metabolic genes between subtypes. **b** Lasso regression analysis of metabolic genes after random survival forest analysis. **c** Multivariate Cox analysis of metabolic genes. **d** Expression heat map between subtypes with the final screened target metabolic genes. **e**–**h** Prognostic model for overall survival, progression-free survival, and relapse-free survival in TCGA cohort, and for progression-free survival in the E-MTAB-4321 cohort. **i**–**l** Receiver operating characteristic curve corresponding to the overall survival, progression-free survival, and relapse-free survival in the prognostic model for TCGA cohort, and progression-free survival in the E-MTAB-4321 cohort

Among the 289 characteristic genes of the metabolic subtypes, random survival forest identified 39 genes with relative importance  ≥ 0 as the final features, and univariate Cox analysis narrowed the list down further to 21 genes with prognostic significance, which was confirmed in lasso regression analysis. Of these 21 genes, 13 remained significant in the multivariate regression model. Samples were divided into high-risk and low-risk groups according to the median expression level of the risk score, and Kaplan–Meier analysis showed significant differences in survival between the groups in TCGA and validation sets. The prognostic accuracy of our signature was further verified based on receiver operating characteristic curve analysis, with areas under the curve > 0.63 for 1-, 3-, and 5-year OS, PFS, or relapse-free survival of the metabolic-related characteristic risk scores of the three cohorts (Fig. 8 and Additional file 5: Figure S5).

## Discussion

Bladder cancer is one of the most commonly reported tumors, and a major contributor to morbidity and mortality worldwide. Staging and grade largely determine the treatment of bladder cancer, and considerably affect the prognosis [23]. Non-MIBC is usually treated by transurethral resection of the bladder tumor and via Bacillus Calmette-Guerin immunotherapy. However, more aggressive treatment methods are necessary for MIBC, including radical cystectomy combined with chemotherapy [23]. Although the use of chemotherapy in neoadjuvant and assistive settings can improve outcomes, the adoption rate remains low [24]. This may result in part from the fact that 50% of all patients exhibit natural resistance to cisplatin-based chemotherapy, and a significant proportion of patients who meet the criteria for cisplatin treatment eventually develop chemoresistance during treatment. Checkpoint inhibitors represent a recently approved second-line drug option with the potential to change the prospects of bladder cancer treatment. Unfortunately, only 20%–30% of the patients show a clinical response to immunotherapy, and long-term data indicate that disease-specific survival rates have not improved [25]. Therefore, further investigation of the molecular characteristics of bladder cancer is essential for the development of tumor-specific targeted drugs.

Based on the analysis of metabolic expression profiles, we propose a new molecular classification method for bladder cancer. Metabolism is considered one of the key characteristics of cancer. Cancer cells tend to use glycolysis as an alternative to the aerobic cycle (oxidative phosphorylation) of normal cells, and therefore use the mitochondria differently, which is known as the Warburg effect [26]. Therefore, clarification of the mitochondrial processes and mechanisms for regulating the aerobic cycle and glycolysis have been an important focus in the research of bladder tumors. The three metabolic subtypes were found to be enriched in drug metabolism-cytochrome P450 and metabolism of xenobiotics by cytochrome P450. Cytochrome P450 (P450) enzymes are important in the metabolism of drugs, steroids, fat-soluble vitamins, carcinogens, pesticides, and many other types of chemicals. Their catalytic activity is an important aspect in fields such as drug-drug interactions and endocrine function. In vitro assays can now be performed to determine which P450s (and other enzymes) participate in the clearance of new drug candidates, thereby predicting drug clearance parameters, drug interactions, and changes in patient-related problems between individuals [27]. Knowledge of the structure of P450 is crucial not only for understanding and predicting drug metabolism but also for investigating its genetic variations, especially in P450 17A1 and 21A2. Revealing the molecular nature of these defects should help to better understand the loss of function and predict the effects of new mutations [28].

Through this comprehensive characterization of the metabolic subtypes with a multi-omics approach, we found that compared with the M1 and M3 subtypes, the M2 subtype participates in the most distinct metabolic pathways, showing the strongest extent of immune infiltration and hypoxia, and the worst prognosis. The abnormal metabolic characteristics of the M2 subtype are mainly manifested in that the end products of glycolysis in the corresponding patient subgroups are significantly upregulated, and their pyruvate metabolism and tricarboxylic acid cycle are significantly activated. In addition, compared with the other subtypes, the M2 subtype was more closely related to the activation of HIF-1A and the tumor hypoxic microenvironment. Several studies have shown that HIF-1A is stabilized in the hypoxic tumor environment owing to the lack of molecular oxygen, which leads to the expression of HIF-1A target genes that in turn activate the expression of many genes involved in glucose metabolism. Abnormal activation of HIF-1A and related metabolic target genes in the M2 subtype not only indicates a response to hypoxia-induced therapy but could also be used as a prognostic biomarker for bladder cancer patients. Consistent with the above observations, our drug sensitivity analysis showed that patients with M2 bladder cancer were most sensitive to the first-line diabetes drug metformin. Patients with type II diabetes have an increased risk of bladder cancer and a poor prognosis [29, 30]. In addition to lowering blood sugar, metformin has been reported to prolong patient life and improve prognosis in a variety of cancers. Experimental results on bladder cancer showed that metformin can exert anti-tumor effects by inhibiting cell proliferation and the stemness signal axis (such as Akt and ERK) [31]. Based on these previous findings, our results strongly suggest the potential clinical value of the M2 subtype and the potential therapeutic feasibility of inhibiting the accompanying pathways.

This study further provides new insight into the relationship between the metabolic classification of bladder cancer and treatment response. With respect to immunotherapy response prediction, although M2 showed the highest immune infiltration state, it had the worst response to treatment based on immune checkpoint inhibitors. High immune infiltration is typically associated with a good prognosis; however, the data for bladder cancer appears to conflict with this general relationship, with no clear explanation put forth to date. Answers to this mystery can be related to recent studies that have revealed two different tumor immune evasion mechanisms. In some tumors, although the degree of cytotoxic T cell infiltration is high, these T cells are often in a state of dysfunction. However, in other tumors, immunosuppressive factors can remove T cells infiltrating the tumor tissue. Jiang et al. [22] designed a new computing architecture, the TIDE score, to integrate these two tumor immune escape mechanisms, which can serve as a substitute for a single marker to effectively predict the effect of immune checkpoint suppression therapy. Thus, although M2 showed the highest immune infiltration state, cytotoxic T cells infiltrating the tumor tissue may be in a dysfunctional state, and these ‘cell police’ would not be able to exert their function in controlling tumor growth. Indeed, the tumor immune dysfunction score of the M2 subtype was consistent with the characteristics of tumor immune escape. In addition, immunotherapy predictions showed the lowest response for the M2 subtype. Together, these findings can explain why the M2 subtype with high immune infiltration also showed the worst prognosis.

By contrast, the M1 subtype shows promise for treatment with immune checkpoint inhibitors. In TCGA cohort, the predicted response rate of M1 to immunotherapy was 61·1%, which was much higher than the 32.9% for M2 and 33.3% for M3. It is expected that further identification of the differences in molecular signaling pathways for different metabolic subtypes may provide useful insights for revealing the relationship between metabolic regulation and tumor immune escape. However, the M1 subtype was predicted to be the type that would most likely exhibit chemotherapy resistance, which remains the main challenge in the treatment of bladder cancer. Up to 50% of the patients do not respond to cisplatin-based chemotherapy, and many of these patients will develop chemoresistance under treatment. Changes in metabolism have proven to fundamentally change the efficacy of drugs. The mitochondria play a central role in drug-induced cell death in the important centers and organelles of tumor cells [32, 33]. Proteins present in the mitochondria, such as cytochrome c, are essential to activate caspase [34–36]. As mentioned previously, all three metabolic subtypes of bladder cancer were found to be enriched in drug metabolism-cytochrome P450 and metabolism of xenobiotics by cytochrome P450; however, compared to M2 and M3, M1 showed natural drug resistance to cisplatin (enriched in platinum drug resistance). Chemosensitivity prediction also verified this difference, as patients with the M1 subtype were the least sensitive to cisplatin. Among the top 20 genes of each subtype, *TP53, PIK3CA, ERBB2*, and *ATM* participate in platinum drug resistance. Among them, *PIK3CA* showed similar mutation frequencies in the three subtypes, whereas the frequency of *TP53* mutation was much higher in the M2 subtype (60%) than that in M1 (40%) and M3 (38%). Mutations in *ERBB2* (14%) and *ATM* (17%) were mainly distributed in the M2 and M3 subtypes, respectively. These results indicate that there are important genes involved in M1 platinum drug resistance, and further research is needed to clarify the mechanism.

Based on the potential clinical value and therapeutic feasibility of metabolic typing, we further developed a signature composed of 13 metabolic genes, which showed excellent performance in predicting the prognosis of bladder cancer. The genes in this signature were not only differentially expressed between the metabolic subtypes of bladder cancer but were also significantly related to the patients’ prognosis in terms of OS, PFS, and relapse-free survival. High risk scores indicated a poor prognosis for bladder cancer patients, which is expected to be applied in actual clinical settings.

## Conclusions

Based on analyses of multiple datasets with multi-omics data, we established a molecular classification of bladder cancer based on metabolism-related subtypes, and more comprehensively characterized the subtypes’ metabolic characteristics, prognostic characteristics, clinical characteristics, immune infiltration, genetic changes, and responses to chemotherapy and immunotherapy. Given that the samples available for bladder cancer classification based on metabolic profiles were limited, analysis with a larger sample size and further basic experiments are needed to support our pioneering classification. Nevertheless, the present in-depth analysis of metabolism can provide a valuable reference and insight to inform the development of new strategies for the precise treatment of bladder cancer.

## Supplementary Information

**Additional file 1:** **Figure S1.** Heatmap of clustering of bladder cancer in TCGA cohort for different subtype numbers (k = 2–6) with NMF.

**Additional file 2:** **Figure S2.** Heatmap of subtype-specific genes and its functional enrichment analysis. Heatmap of **a** up-expressed and **b** down-expressed genes among three subtypes. GO and KEGG items for **c**, **d** M1, **e**, **f** M2 and **g**, **h** M3. *BP* biological process, *CC* cellular component, *MF* molecular function, *GO* Gene Ontology, *KEGG* Kyoto Encyclopedia of Genes and Genomes.

**Additional file 3:** **Figure S3.** Functional enrichment analysis for top 20 genes in samples of each subtype. The top-level Gene Ontology biological processes for **a**–**c** M1, M2 and M3. Network of enriched terms for **d**–**f** M1, M2 and M3. Protein–protein interaction network of hub genes among **g**–**i** M1, M2 and M3. Summary of enrichment analysis in DisGeNET for **j**–**l** M1, M2 and M3.

**Additional file 4:** **Figure S4**. Chromosomal aberrations among three subtypes. **a-c** Amplification and **d**-**e** deletion for M1, M2 and M3.

**Additional file 5:** **Figure S5.** Prognostic risk model based on characteristic genes of the metabolic subtypes in other validation cohorts. . **a-c** Prognostic model for overall survival in the GSE13507, GSE31684 and GSE32548 cohorts, **d**–**f** receiver operating characteristic curve corresponding to the overall survival of GSE13507, GSE31684 and GSE32548 cohorts.

**Additional file 6: Table S1.** Correlation between metabolic subclasses and clinical features of bladder cancer patients in TCGA cohort. **Table S2.** Correlation between metabolic subclasses and clinical features of bladder cancer patients in GSE32894 cohort. **Table S3.** Correlation between metabolic subclasses and clinical features of bladder cancer patients in E-MTAB-4321 cohort. **Table S4.** The specific P-values of drugs among metabolic subclasses.

## Data Availability

The datasets generated or analyzed for this study can be found in the TCGA Knowledge Base (https://portal.gdc.cancer.gov/repository), GEO (https://www.ncbi.nlm.nih.gov/geo/), and ArrayExpress (https://www.ebi.ac.uk/arrayexpress/) databases.

## Abbreviations

MIBC: Muscle-invasive bladder cancer
RNA-seq: RNA-sequencing
TCGA: The Cancer Genome Atlas
GEO: Gene Expression Omnibus
NMF: Non-negative matrix factorization
ssGSEA: Single-sample gene set enrichment analysis
GSVA: Gene set variation analysis
TIDE: Tumor Immune Dysfunction and Rejection
GDSC: Genomics of Cancer Drug Sensitivity
IC50: Half-maximal inhibitory concentration
OS: Overall survival
PFS: Progression-free survival
