# Supplementary figures and images for: Metabolic classification of bladder cancer based on multi-omics integrated analysis to predict patient prognosis and treatment response
Source: J Transl Med. 2021 May 13;19:205. doi: 10.1186/s12967-021-02865-8 (PMC8117567; doi:10.1186/s12967-021-02865-8)

rank = 2

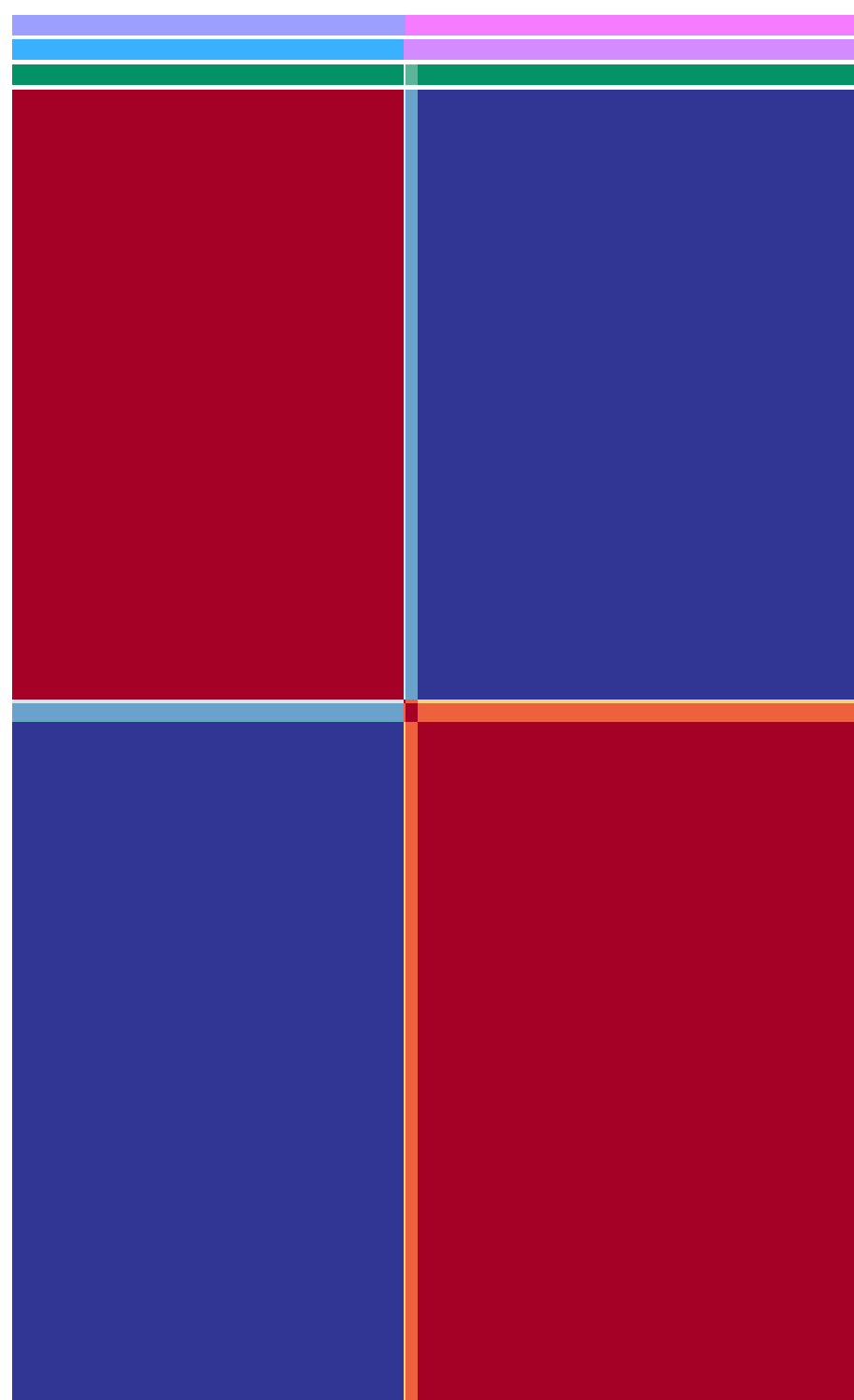

rank = 3

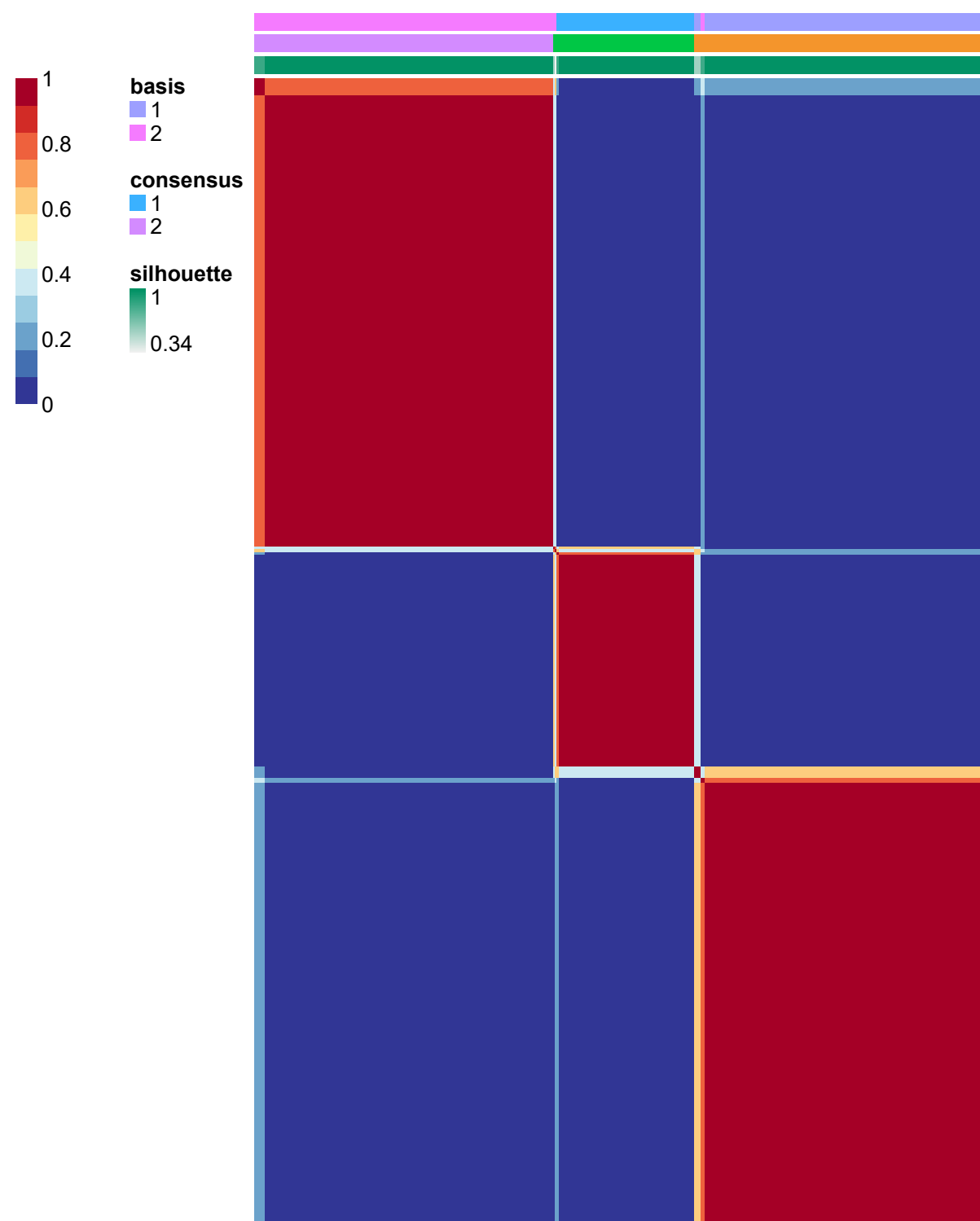

rank = 4

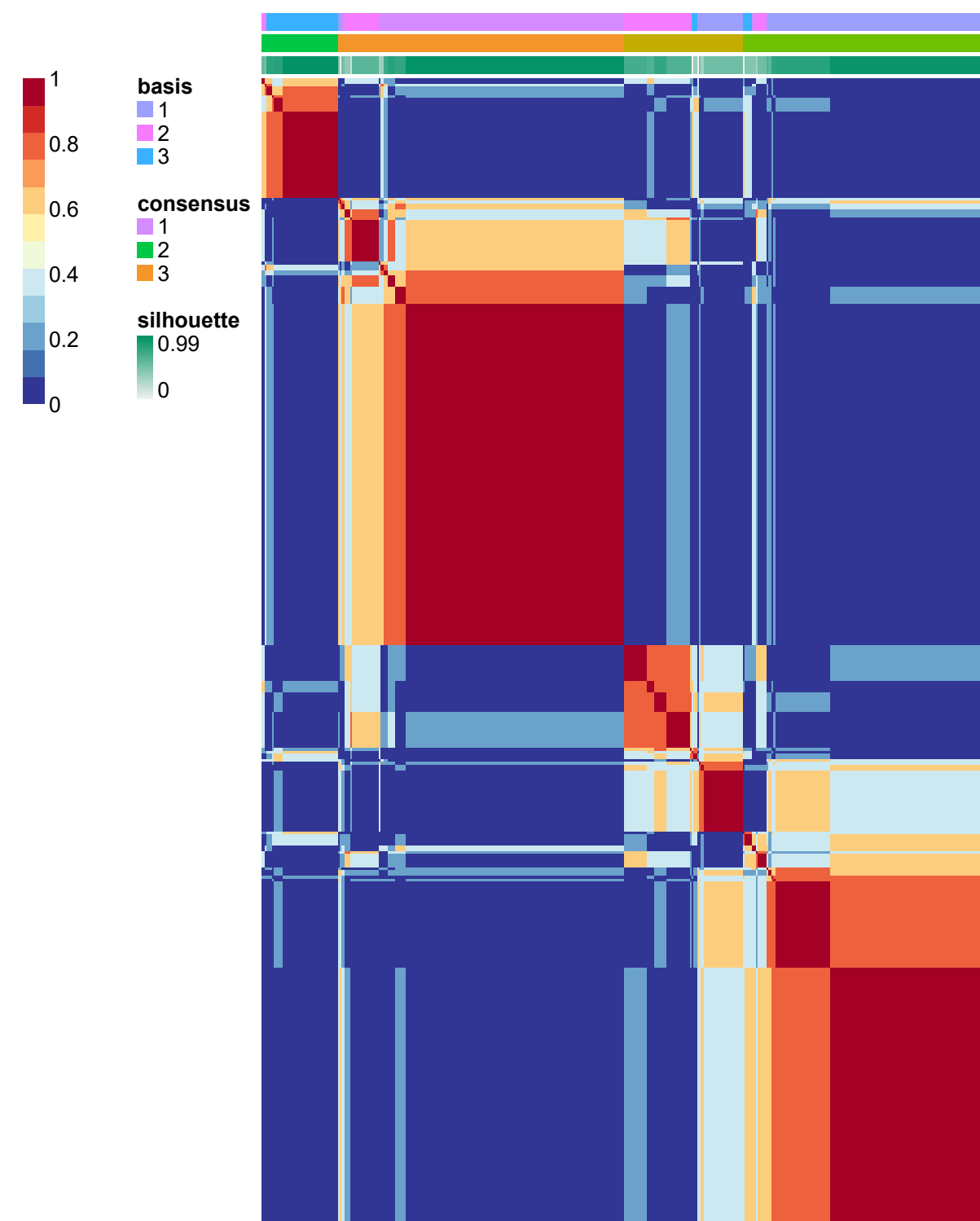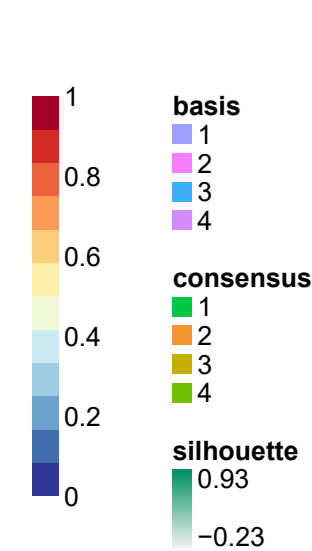

rank = 5

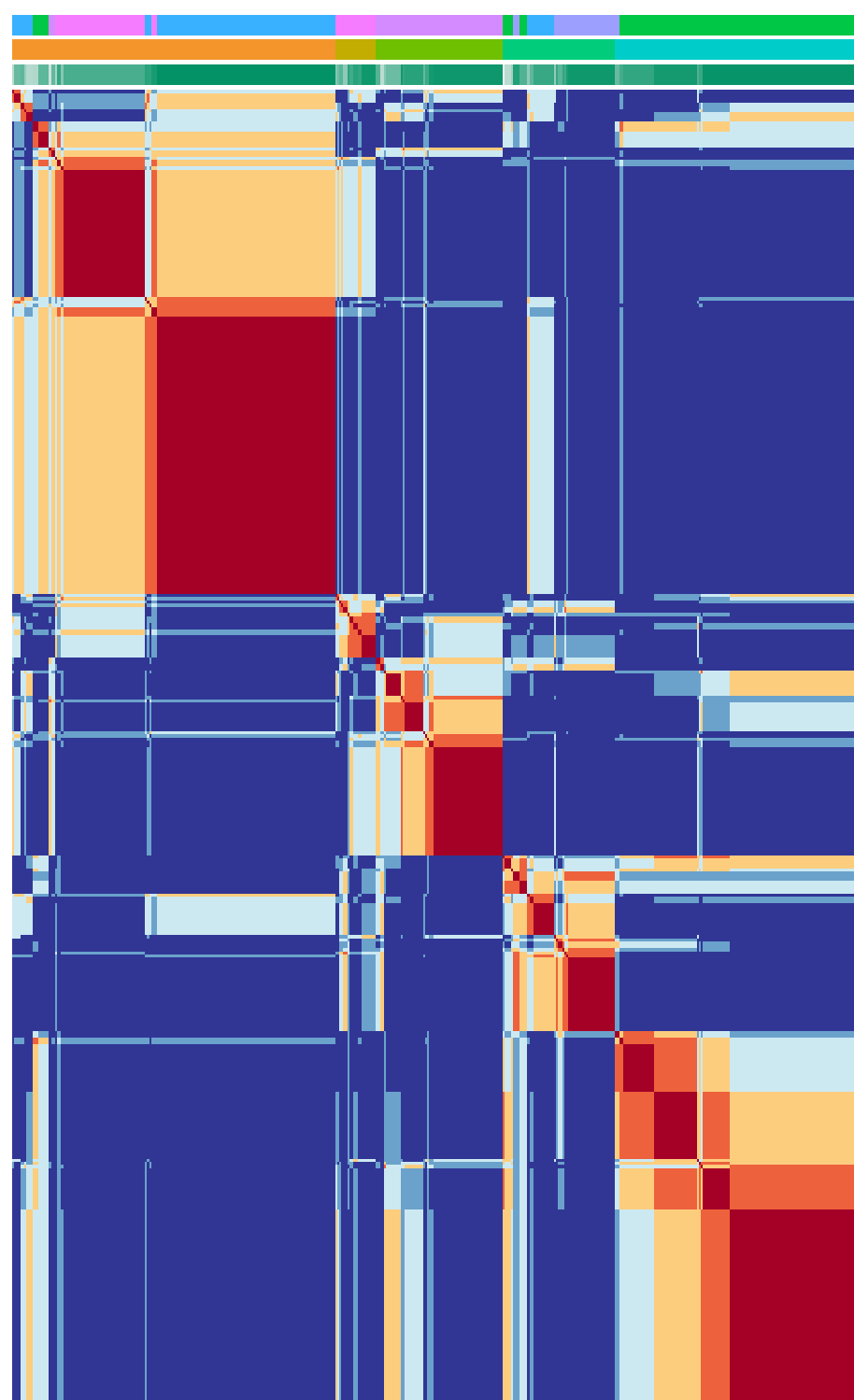

rank = 6

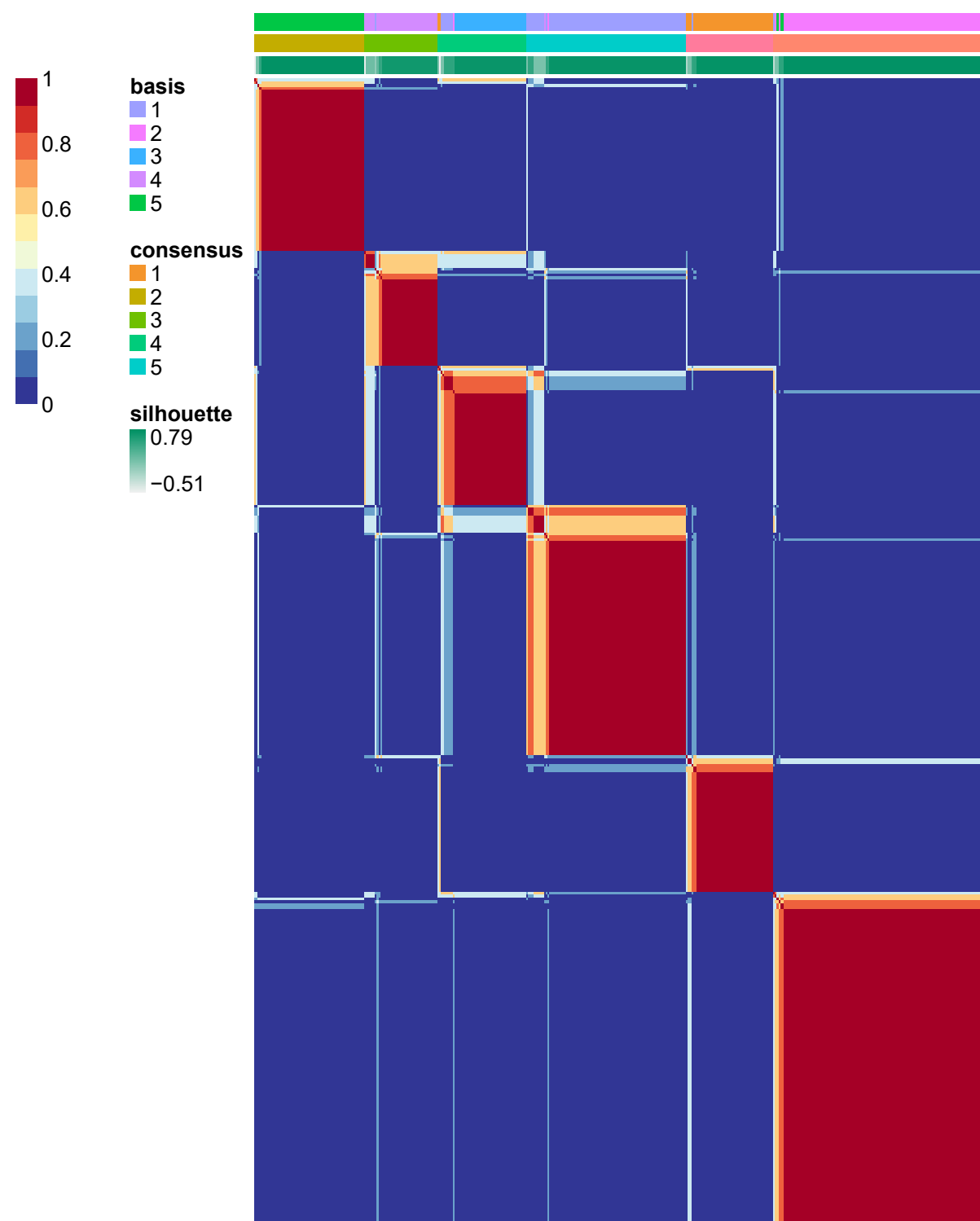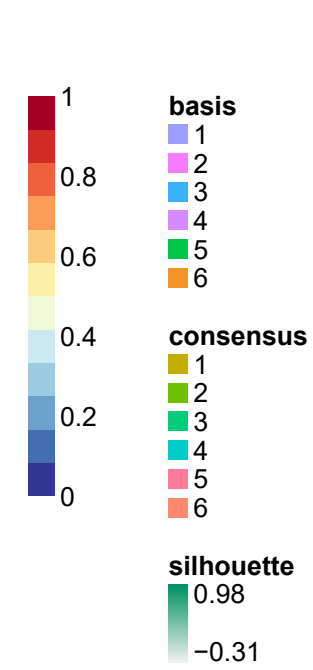

Supplement: Supplementary file 1 — Additional file 1: Figure S1. Heatmap of clustering of bladder cancer in TCGA cohort for different subtype numbers (k = 2–6) with NMF. [file 12967_2021_2865_MOESM1_ESM.pdf]

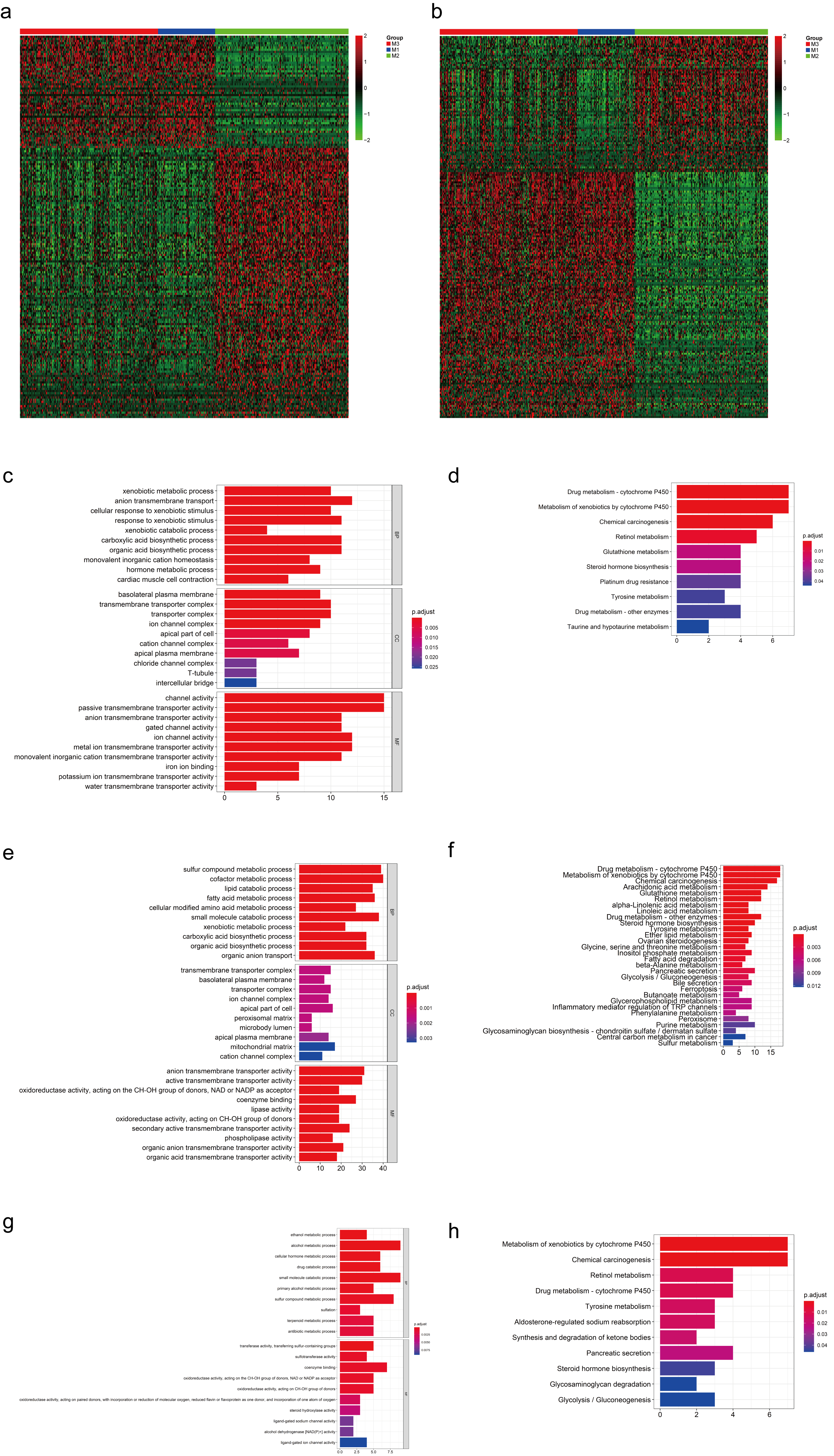

Supplement: Supplementary file 2 — Additional file 2: Figure S2. Heatmap of subtype-specific genes and its functional enrichment analysis. Heatmap of a up-expressed and b down-expressed genes among three subtypes. GO and KEGG items for c, d M1, e, f M2 and g, h M3. BP biological process, CC cellular component, MF molecular function, GO Gene Ontology, KEGG Kyoto Encyclopedia of Genes and Genomes. [file 12967_2021_2865_MOESM2_ESM.tif]

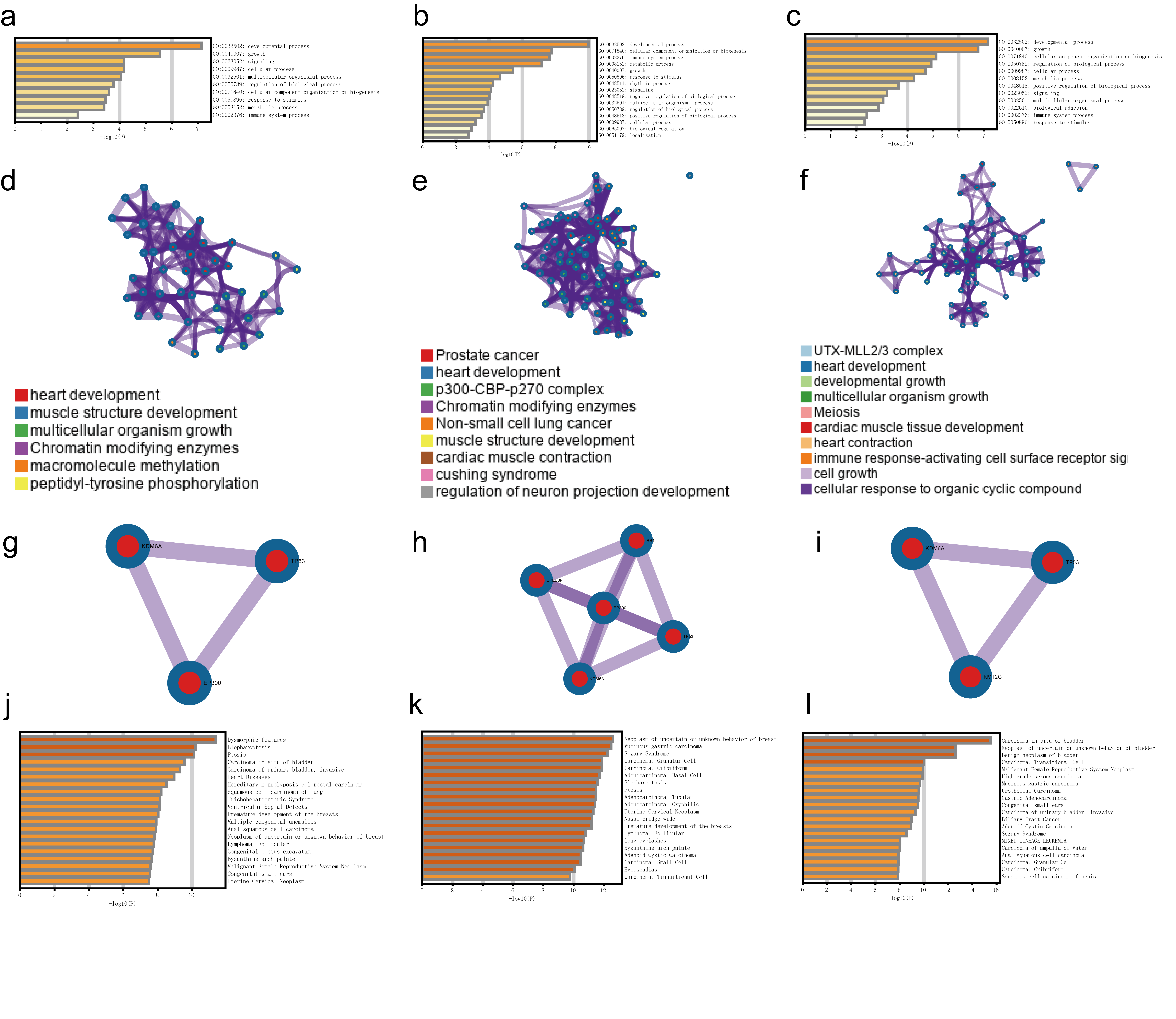

Supplement: Supplementary file 3 — Additional file 3: Figure S3. Functional enrichment analysis for top 20 genes in samples of each subtype. The top-level Gene Ontology biological processes for a–c M1, M2 and M3. Network of enriched terms for d–f M1, M2 and M3. Protein–protein interaction network of hub genes among g–i M1, M2 and M3. Summary of enrichment analysis in DisGeNET for j–l M1, M2 and M3. [file 12967_2021_2865_MOESM3_ESM.tif]

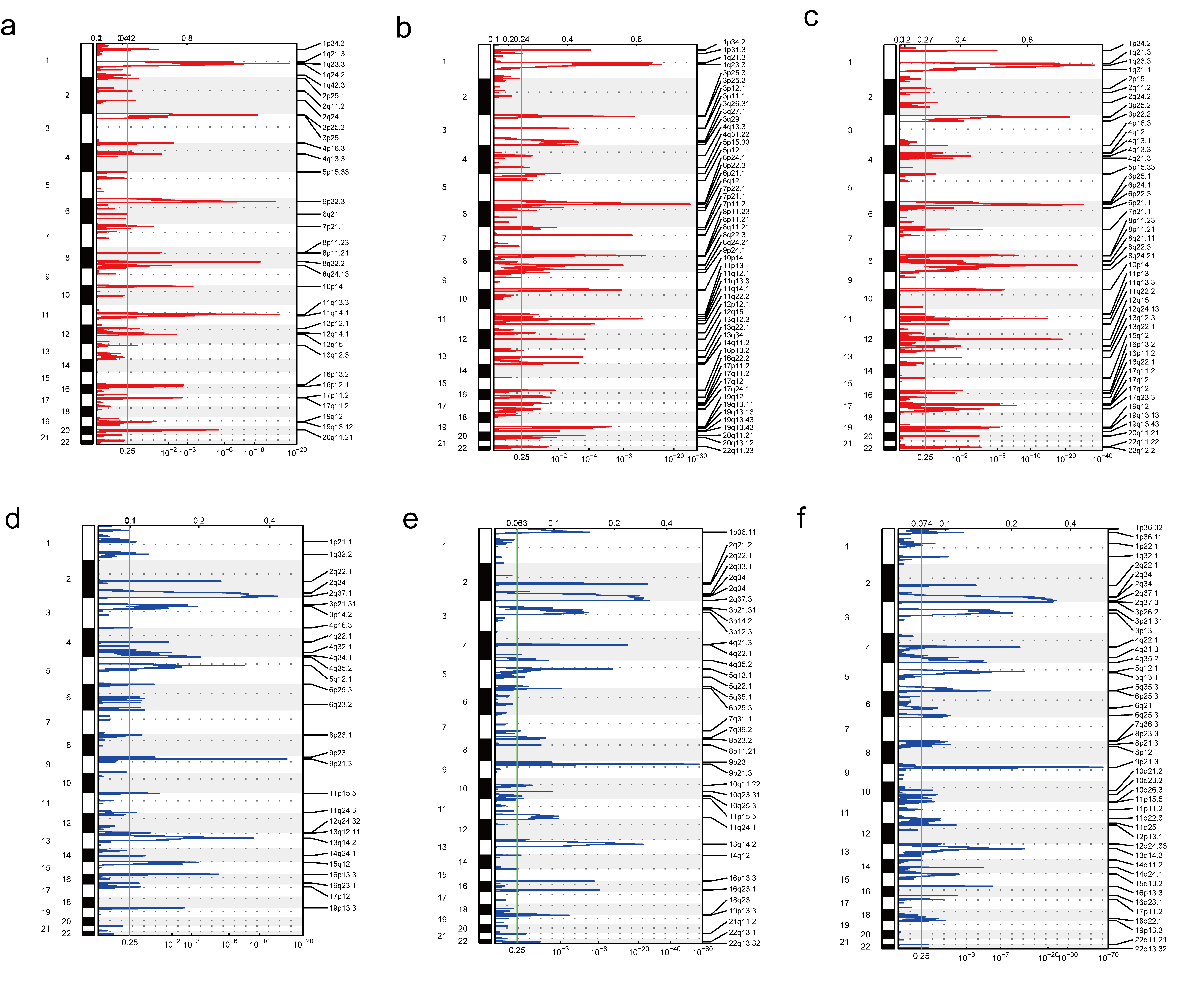

Supplement: Supplementary file 4 — Additional file 4: Figure S4. Chromosomal aberrations among three subtypes. a-c Amplification and d-e deletion for M1, M2 and M3. [file 12967_2021_2865_MOESM4_ESM.tif]

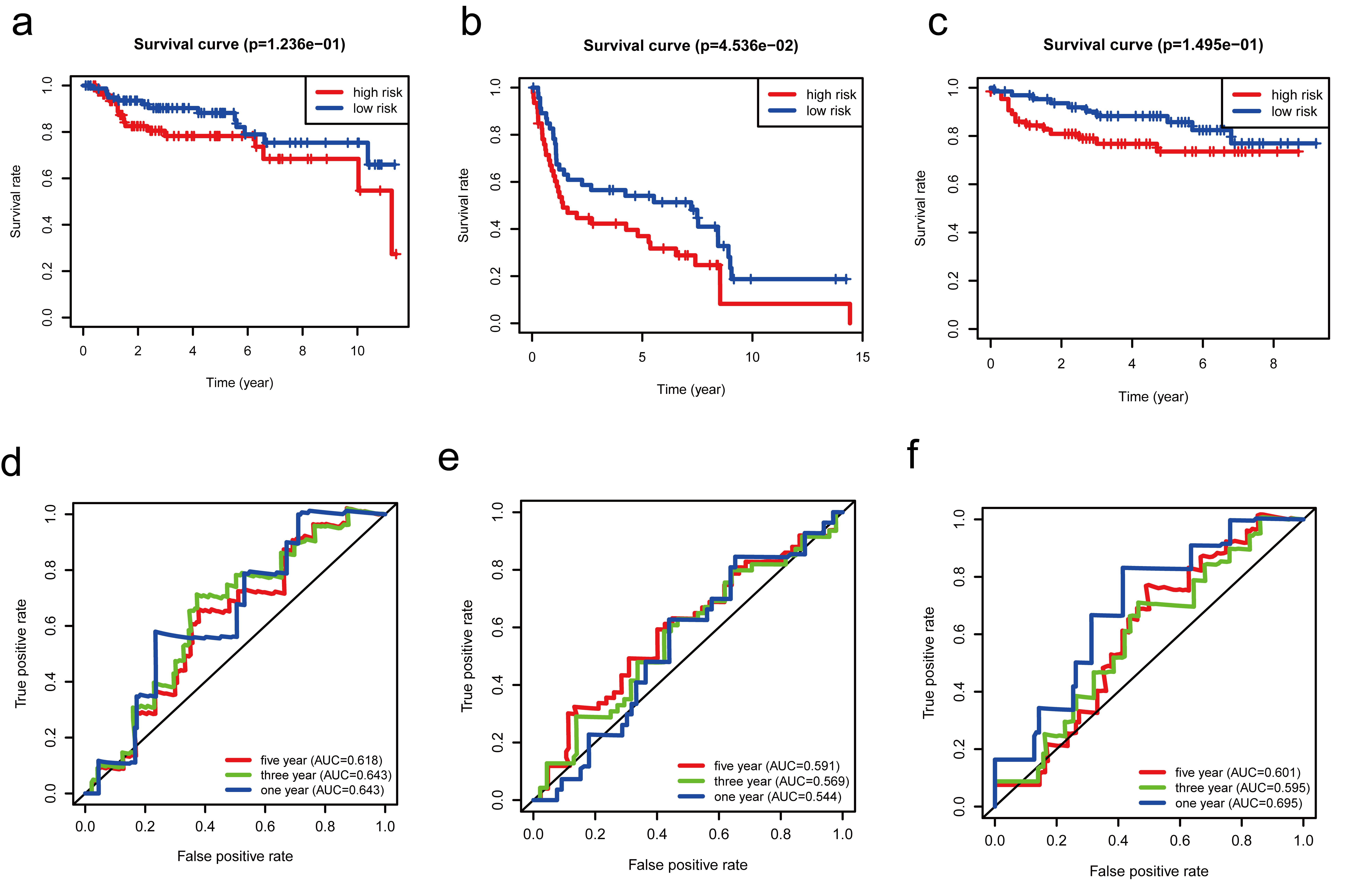

Supplement: Supplementary file 5 — Additional file 5: Figure S5. Prognostic risk model based on characteristic genes of the metabolic subtypes in other validation cohorts. . a-c Prognostic model for overall survival in the GSE13507, GSE31684 and GSE32548 cohorts, d–f receiver operating characteristic curve corresponding to the overall survival of GSE13507, GSE31684 and GSE32548 cohorts. [file 12967_2021_2865_MOESM5_ESM.tif]
