# Supplementary material for: Metabolic classification of bladder cancer based on multi-omics integrated analysis to predict patient prognosis and treatment response
Source: J Transl Med. 2021 May 13;19:205. doi: 10.1186/s12967-021-02865-8 (PMC8117567; doi:10.1186/s12967-021-02865-8)
Supplement: Supplementary file 6 — Additional file 6: Table S1. Correlation between metabolic subclasses and clinical features of bladder cancer patients in TCGA cohort. Table S2. Correlation between metabolic subclasses and clinical features of bladder cancer patients in GSE32894 cohort. Table S3. Correlation between metabolic subclasses and clinical features of bladder cancer patients in E-MTAB-4321 cohort. Table S4. The specific P-values of drugs among metabolic subclasses. [file 12967_2021_2865_MOESM6_ESM.docx]

Supplementary Table 1. Correlation between metabolic subclasses and clinical features of bladder cancer patients in TCGA cohort.

| Clinical parameters | | total | M1 | M2 | M3 | P-value | FDR |
| --- | --- | --- | --- | --- | --- | --- | --- |
|  |  | n=408 | n=69 | n=165 | n=174 |  |  |
| gender | MALE | 301 | 57 | 112 | 132 | 0.0464 | 0.0619 |
|  | FEMALE | 107 | 12 | 53 | 42 |  |  |
| age | <65 | 150 | 27 | 61 | 62 | 0.8759 | 0.8956 |
|  | ≥65 | 258 | 42 | 104 | 112 |  |  |
| tumor grading | low grade | 21 | 9 | 1 | 11 | 0.0009 | 0.0038 |
|  | high grade | 384 | 59 | 164 | 161 |  |  |
|  | unknow | 3 | 1 | 0 | 2 |  |  |
| stage | Stage I | 2 | 1 | 0 | 1 | 0.0181 | 0.0362 |
|  | Stage II | 130 | 32 | 40 | 58 |  |  |
|  | Stage III | 140 | 15 | 69 | 56 |  |  |
|  | Stage IV | 134 | 20 | 56 | 58 |  |  |
|  | unknow | 2 | 1 | 0 | 1 |  |  |
| T | T0 | 1 | 1 | 0 | 0 | 0.0016 | 0.0044 |
|  | T1 | 3 | 1 | 1 | 1 |  |  |
|  | T2 | 119 | 33 | 37 | 49 |  |  |
|  | T3 | 194 | 21 | 96 | 77 |  |  |
|  | T4 | 58 | 8 | 21 | 29 |  |  |
|  | unknow | 33 | 5 | 10 | 18 |  |  |
| M | M0 | 196 | 38 | 63 | 95 | 0.0244 | 0.0390 |
|  | M1 | 11 | 1 | 5 | 5 |  |  |
|  | M_X_ or unknow | 201 | 30 | 97 | 74 |  |  |
| N | N0 | 237 | 41 | 96 | 100 | 0.8956 | 0.8956 |
|  | N1-3 | 129 | 19 | 53 | 57 |  |  |
|  | N_X_ or unknow | 42 | 9 | 16 | 17 |  |  |
| molecular subtype | Luminal | 26 | 5 | 0 | 21 | 1.59E-54 | 1.27E-53 |
|  | Luminal infiltrated | 78 | 12 | 14 | 52 |  |  |
|  | Luminal papillary | 142 | 50 | 9 | 83 |  |  |
|  | Basal squamous | 142 | 2 | 129 | 11 |  |  |
|  | Neuronal | 20 | 0 | 13 | 7 |  |  |

T, tumor; M, metastasis; N, lymph node. P-values were obtained by Fisher’s exact test; FDR was corrected by the Benjamini & Hochberg methods.

Supplementary Table 2. Correlation between metabolic subclasses and clinical features of bladder cancer patients in GSE32894 cohort.

| Clinical parameters | | total | M1 | M2 | M3 | P-value | FDR |
| --- | --- | --- | --- | --- | --- | --- | --- |
|  |  | n=308 | n=121 | n=80 | n=107 |  |  |
| sex | female | 80 | 38 | 27 | 15 | 0.0021 | 0.0026 |
|  | male | 228 | 83 | 53 | 92 |  |  |
| age | <65 | 88 | 39 | 19 | 30 | 0.4231 | 0.4231 |
|  | ≥65 | 220 | 82 | 61 | 77 |  |  |
| Tumor Stage | Ta | 116 | 83 | 6 | 27 | 3.22E-28 | 8.06E-28 |
|  | T1 | 97 | 27 | 16 | 54 |  |  |
|  | T2-4 | 93 | 10 | 58 | 25 |  |  |
|  | Tx | 2 | 1 | 0 | 1 |  |  |
| Tumor Grade | G1 | 48 | 42 | 2 | 4 | 6.97E-24 | 1.16E-23 |
|  | G2 | 103 | 59 | 9 | 35 |  |  |
|  | G3-4 | 155 | 20 | 67 | 68 |  |  |
|  | Gx | 2 | 0 | 2 | 0 |  |  |
| Molecular Subtype | urobasal A | 131 | 96 | 1 | 34 | 9.12E-49 | 4.56E-48 |
|  | genomical-ly unstable | 85 | 8 | 15 | 62 |  |  |
|  | urobasal B | 20 | 9 | 10 | 1 |  |  |
|  | infiltrated | 43 | 8 | 25 | 10 |  |  |
|  | SCC-like | 29 | 0 | 29 | 0 |  |  |
|  |  |  |  |  |  |  |  |

T, tumor; G, grade. P-values were obtained by Fisher’s exact test; FDR was corrected by the Benjamini & Hochberg methods.

Supplementary Table 3. Correlation between metabolic subclasses and clinical features of bladder cancer patients in E-MTAB-4321 cohort.

| Clinical parameters | | total | M1 | M2 | M3 | P-value | FDR |
| --- | --- | --- | --- | --- | --- | --- | --- |
|  |  | n=476 | n=136 | n=124 | n=216 |  |  |
| tumor grading | low grade | 277 | 73 | 43 | 161 | 2.02E-12 | 1.62E-11 |
|  | high grade | 192 | 61 | 81 | 50 |  |  |
|  | PUNLMP | 7 | 2 | 0 | 5 |  |  |
| disease staging | CIS | 3 | 1 | 1 | 1 | 2.96E-09 | 1.18E-08 |
|  | Ta | 345 | 102 | 66 | 177 |  |  |
|  | T1 | 112 | 32 | 43 | 37 |  |  |
|  | T2-4 | 16 | 1 | 14 | 1 |  |  |
| sex | male | 367 | 113 | 94 | 160 | 0.1353 | 0.1547 |
|  | female | 109 | 23 | 30 | 56 |  |  |
| age | <65 | 167 | 41 | 37 | 89 |  |  |
|  | ≥65 | 309 | 95 | 87 | 127 | 0.0387 | 0.0516 |
| tumor size | < 3cm | 283 | 86 | 62 | 135 |  |  |
|  | >= 3cm | 87 | 21 | 35 | 31 | 0.0068 | 0.0109 |
|  | unknown | 106 | 29 | 27 | 50 |  |  |
| Histologic-al | papillary | 417 | 122 | 94 | 201 | 1.64E-05 | 4.37E-05 |
|  | solid | 14 | 2 | 11 | 1 |  |  |
|  | mixed | 8 | 2 | 5 | 1 |  |  |
|  | unknown | 37 | 10 | 14 | 13 |  |  |
| BCG treatment | no | 388 | 107 | 99 | 182 | 0.2759 | 0.2759 |
|  | yes | 88 | 29 | 25 | 34 |  |  |
| CIS in disease course | no | 402 | 119 | 91 | 192 |  |  |
|  | yes | 74 | 17 | 33 | 24 | 0.0004 | 0.0008 |

BCG, bacillus Calmette-Guérin; CIS, carcinoma in situ; P-values were obtained by Fisher’s exact test; FDR was corrected by the Benjamini & Hochberg method

Supplementary Table 4. The specific P-values of drugs among metabolic subclasses.

| Drugs | P-value |
| --- | --- |
| Bleomycin | 3.40E-52 |
| Cisplatin | 2.47E-17 |
| Doxorubicin | 1.51E-08 |
| Erlotinib | 3.28E-35 |
| Gefitinib | 2.34E-10 |
| Gemcitabine | 1.07E-41 |
| Mitomycin.C | 1.10E-11 |
| Rapamycin | 0.000258222 |
| Sunitinib | 2.84E-25 |
| Embelin | 7.82E-47 |
| Etoposide | 1.22E-21 |
| Imatinib | 5.34E-16 |
| Lapatinib | 9.10E-20 |
| Metformin | 1.03E-36 |
| Paclitaxel | 5.68E-15 |
| Pazopanib | 2.65E-15 |
| Sorafenib | 2.72E-15 |
| Temsirolimus | 3.70E-46 |
| Vinblastine | 7.87E-33 |
| Vinorelbine | 1.85E-06 |
| VX.680 | 6.91E-23 |
| Methotrexate | 4.43E-05 |
